# Predictive value of estimated plasma volume for postoperative hypotension in percutaneous intramyocardial septal radiofrequency ablation treating for hypertrophic obstructive cardiomyopathy

**DOI:** 10.1186/s12872-024-03844-9

**Published:** 2024-03-22

**Authors:** Bo Shan, Jing Li, Zhangwei Shi, Chao Han, Juan Zhang, Jia Zhao, Rui Hu, Liwen Liu, Shengjun Ta

**Affiliations:** 1 Department of Ultrasound, Hypertrophic Cardiomyopathy Center, Xijing Hospital, Air Force Medical University, Xi’an, Shaanxi China; 2 Department of Cardiology, Xijing Hospital, Air Force Medical University, Xi’an, Shaanxi China

**Keywords:** Hypertrophic cardiomyopathy, Percutaneous intramyocardial septal radiofrequency ablation, Hypotension, Estimated plasma volume status

## Abstract

**Background:**

Estimated plasma volume status (ePVS) estimated by the Duarte formula is associated with clinical outcomes in patients with heart failure. It remains unclear the predictive value of the ePVS to the postoperative hypotension (POH) in percutaneous intramyocardial septal radiofrequency ablation (PIMSRA) treating hypertrophic obstructive cardiomyopathy (HOCM).

**Methods:**

Data of HOCM patients who underwent PIMSRA were retrospectively collected. Preoperative ePVS was calculated using the Duarte formulas which derived from hemoglobin and hematocrit ratios. Clinical variables including physical assessment, biological and echocardiographic parameters were recorded. Patients were labeled with or without POH according to the medical record in the hospital. Univariable and multivariable logistic regression were performed to evaluate the association between ePVS and POH. Using different thresholds derived from quartiles and the best cutoff value of the receiver operating characteristic curve, the diagnostic performance of ePVS was quantified.

**Results:**

Among the 405 patients included in this study, 53 (13.1%) patients were observed with symptomatic POH. Median (IQR) of ePVS in overall patients was 3.77 (3.27~4.40) mL/g and in patients with POH were higher than those without POH. The ePVS was associated with POH, with the odds ratio of 1.669 (95% CI 1.299 ~ 2.144) per mL/g. After adjusted by potential confounders, ePVS remained independently associated with POH, with the approximate odds ratio in different models.

**Conclusion:**

The preoperative ePVS derived from the Duarte formulas was independently associated with postoperative hypotension in HOCM patients who underwent PIMSRA and showed prognostic value to the risk stratification of postoperative management.

**Trial registration:**

NCT06003478 (22/08/2023).

**Supplementary Information:**

The online version contains supplementary material available at 10.1186/s12872-024-03844-9.

## Background

Hypertrophic obstructive cardiomyopathy (HOCM) is mainly characterized by left ventricular outflow tract (LVOT) obstruction due to unexplained myocardial hypertrophy and septal reduction therapies are considered the preferred treatments for HOCM patients with drug-refractory symptom [1, 2]. Most patients benefited from the amelioration of LVOT obstruction and the improvement of myocardial contractile function in the follow-up period [3–5]. As an innovative procedure for the treatment of HOCM, PIMSRA has been clinically performed for several years and several studies have confirmed its safety and effectiveness in the majority of cases [3, 4]. In a small number of cases, whereas, the occurrence of postoperative hypotension (POH), which could aggravate severe LVOT obstruction and consequent low cardiac output, remains a challenging complication during perioperative management. In addition, plenty of studies authenticate that POH is associated with an increased risk of long-term prognosis [6]. Therefore, ascertaining the risk factors of POH and preoperatively identifying patients with a high risk of POH is of great significance for optimizing the management of PIMSRA.

Studies found that some indicators reflecting patients’ demographic, hemodynamic, and metabolic profiles may predict the risk of postoperative hypotension, and hypotension can be subcategorized as cardiogenic or hypovolemic in most cases [7, 8]. The estimated plasma volume status (ePVS) estimated by the Duarte formula which assesses the volume overload and reflects the prognosis at a very low cost, has proven to be associated with clinical outcomes in patients with heart failure (HF) [9–11]. Several reports assessed the correlation between ePVS and actual plasma volume measured using conventional radioisotope-labeled albumin or red blood cell assays and found that the ePVS derived from the formulas and actual plasma volume using the gold standard method showed a moderate-to-strong correlation [12–14]. To date, no researchers have evaluated ePVS and analyzed its association with prognosis in hypertrophic cardiomyopathy.

This study intended to analyze the ePVS in patients with hypertrophic cardiomyopathy and analyze whether it is related to postoperative hypotension after PIMSRA treatment.

## Methods

### Study population

We performed this retrospective analysis (NCT06003478 [22/08/2023]) of patients prospectively enrolled in the registry trials of PIMSRA (NCT02888132 & NCT04355260) and the primary results were published earlier [3, 4]. Data on consecutive patients who were diagnosed with HOCM followed by the 2023 ESC Guidelines for the management of cardiomyopathies and had undergone PIMSRA at the Xijing Hospital between October 2016 and December 2022 were retrospectively collected. We excluded 3 patients with preoperative hypotension (mean systolic blood pressure (SBP) < 90 mmHg preoperatively measured by ambulatory blood pressure monitoring). Informed consent for the procedure and clinical research was obtained from all individual participants before procedure. Informed consent for their data to be used for research was obtained from all patients before they received PIMSRA. This retrospective analysis was based on anonymous data and the exception to the requirement of informed consent was approved by the Institutional Ethics Committee of the Xijing Hospital. Research procedures followed the amended Helsinki Declaration of 1975. The study was approved by the Institutional Ethics Committee of the Xijing Hospital.

### Collection of baseline characteristics and calculation of estimated plasma volume status

Information of medical history was collected from the admission note and the family history was collected from the latest follow-up records. Laboratory blood tests, transthoracic echocardiogram examinations, 12-lead electrocardiograms, computed tomography angiography, ambulatory blood pressure monitoring, and Holter electrocardiographic monitoring were performed perioperatively. The blood test was performed within one week before PIMSRA by our research protocol. Baseline blood pressures and heart rates were recorded using daytime mean blood pressures preoperatively obtained from ambulatory blood pressure monitoring, and missing data were filled by results of routine blood pressure measurement at admission. Mitral regurgitation and systolic anterior motion of the mitral valve were evaluated by echocardiogram following the established classification methods [15, 16]. The results were collected from the Hospital Information System of Xijing Hospital. ePVS was calculated using hematocrit and hemoglobin from the Strauss-derived Duarte formula as follows [9]:

$$ePVS(mL/g)=1000\times (1-hematocrit)/hemoglobin(g/L)$$

Information of procedure duration time was acquired from the operation note and calculated as total working time of ablation.

### Definition of postoperative hypotension

A consensus statement on postoperative blood pressure was provided in the Perioperative Quality Improvement (POQI) 3 document, which pointed out that a systolic pressure of less than 90 mmHg is likely to put the majority of patients at risk of end-organ damage [17]. This criterion is also consistent with the definitions used by the majority of researchers in earlier investigations of perioperative hypotension [18]. Based on these, our study defined patients with postoperative hypotension (POH) as follows: regardless of the dosage of vasopressor agents utilized, recording at least once the SBP less than 90 mmHg obtained by invasive or noninvasive blood pressure measurements, with parallel hypotension-related symptoms such as dizziness, nausea, fatigue, palpitations, or syncope, which necessitate adjustment to the dose of vasopressor agents. The Hospital Information System and postoperative care records were analyzed to obtain data of the patient's blood pressure, symptoms, and medicine usage.

### Statistical analysis

Categorical variables were expressed as frequencies (percentages). Several continuous variables cannot be considered to obey the normal distribution in this study so continuous variables were expressed as median (interquartile range, IQR). Logarithmic transformation was applied to N-terminal pro-brain natriuretic peptide (NT-proBNP) concentrations. Missing values were interpolated by multiple interpolation methods. In detail, we used the “mice” package to perform multiple interpolations with the method of predictive-mean matching to produce five imputed data sets. The subsequent analysis is based on a pooled analysis of the results of the imputed data sets. Comparisons of demographic, clinical, biological, and echocardiographic parameters among patients with or without POH were analyzed using Chi-square tests for categorical variables and a two-sided *t*-test or Kruskal–Wallis test for continuous variables. The above analyses were also performed in the groups with lower or higher ePVS. Logistic regression models were used to obtain unadjusted and covariate-adjusted odds ratios (ORs) between ePVS and postoperative hypotension. Covariates used for adjustment included age, sex, NYHA class, baseline log-BNP, baseline medication, creatinine which were used to evaluate the renal function and time duration of the procedure. In addition, variables with significant differences between patients with and without POH were used as candidates for another multivariable logistic regression which was performed using a global optimization search. To determine the cut-off value of ePVS for prediction of the POH risk, receiver operating characteristic (ROC) curve analysis was performed. Effects of ePVS on POH were assessed using both continuous and categorical variables which were transformed by the quartiles and best threshold determined by ROC curve analysis. The forecast capacities of each threshold were calculated. As a sensitive analysis, we also calculated the ePVS derived from the Hakim-Kaplan formula using the measured weight instead of dry weight, which was calculated as follows:

$$Hakim-Kaplan \,ePVS=\frac{aPV-iPV}{iPV}\times 100\%$$

$$aPV=\left(1-hematocrit\right)\times \left(a+b\times weight\right)$$

$$iPV=c\times weight$$

The unit of weight in the formula is kg and the adjustment factors were a = 1,530 in males and 864 in females, b = 41 in males and 47.9 in females, and c = 39 in males and 40 in females. All analyses were performed using R (4.2.3 R Development Core Team, Vienna, Austria). A two-sided *p* < 0.05 was considered statistically significant.

## Results

### Baseline characteristics

The 405 patients had a median (IQR) age of 49 (39~58) years and a total of 150 (37.0%) were women. 243 (60.0%) patients were with severe HF symptoms and diagnosed as NYHA class III or IV. 30 (7.5%) of all patients were taking diuretics and 17 (4.2%) had previously undergone SRT. The median (IQR) of SBP was 116 (109~126) mmHg (Table 1). There were 53 (13.1%) patients suffering from POH. Between patients with or without POH, there were statistical differences in the distribution of sex, BMI, hypertension, and history of syncope or presyncope.

**Table 1:** Clinical and demographic characteristics of patients with or without postoperative hypotension

	Missing n (%)	Patients with POH (*N*=53)	Patients without POH (*N*=352)	*P* Value
Sex, Male	0	22 (41.5)	233 (66.2)	0.001
Age, year	0	49 (37 ~ 61)	49 (40 ~ 57)	0.987
BMI, kg/m^2^	0	23.56 (20.83 ~ 25.39)	25.65 (23.33 ~ 28.08)	<0.001
Diabetes Mellitus	0	3 (5.7)	23 (6.5)	>0.999
Hypertension	0	9 (17.0)	122 (34.7)	0.010
Coronary Heart Disease	6 (1.5)	4 (7.5)	41 (11.8)	0.491
Family History of HCM	0	15 (28.3)	89 (25.3)	0.639
Family History of SCD	0	10 (18.9)	38 (10.8)	0.090
ACEI or ARB	0	6 (11.3)	76 (21.6)	0.083
*β*-blocker	0	50 (94.3)	336 (95.5)	0.992
CCB	0	7 (13.2)	73 (20.7)	0.199
Diuretics	3 (0.7)	4 (7.5)	26 (7.4)	>0.999
Prior SRT	0	4 (7.5)	13 (3.7)	0.349
Syncope or presyncope	0	34 (64.2)	129 (36.6)	<0.001
NYHA III to IV	0	36 (67.9)	207 (58.8)	0.207

Categorical variables were measured with count (percentage) and numerical variables were measured with median (interquartile range)

*BMI* Body mass index, *HCM* Hypertrophic cardiomyopathy, *SCD* Sudden cardiac death, *ACEI* Angiotensin converting enzyme inhibitor, *ARB* Angiotensin receptor blocker, *CCB* Calcium channel blocker, *SRT* Septal reduction therapy, *NYHA* New York Heart Association, *SRT* Septal reduction therapy

All patients who underwent PIMSRA were measured with baseline median (IQR) LVOT pressure gradient of 78 (50~107) mmHg at rest and 123 (92~156) mmHg at provoked peak. Several characteristics measured by echocardiogram and results of lab examinations were significantly different between patients with or without postoperative hypotension. After adjusted with body surface area, the left ventricular volume indexes, both in the end-diastolic and systolic stages, were smaller in patients with POH. There was no significant difference in the degree of myocardial hypertrophy (Table 2).

**Table 2:** Baseline characteristics between patients with or without postoperative hypotension

	Missing n (%)	Patients with POH (*N*=53)	Patients without POH (*N*=352)	*P* Value
SBP, mmHg	0	111 (103 ~ 117)	118 (110 ~ 127)	<0.001
DBP, mmHg	0	64 (60 ~ 69)	69 (62 ~ 75)	0.001
HR, bpm	0	71 (64 ~ 80)	72 (67 ~ 79)	0.806
NSVT	0	8 (15.1)	48 (13.6)	0.774
Atrial Fibrillation	0	4 (7.5)	23 (6.5)	>0.999
LVOTPG at rest, mmHg	0	79 (57 ~ 107)	78 (48 ~ 107)	0.396
LVOTPG provoking, mmHg	6 (1.5)	120 (82 ~ 154)	125 (94 ~ 156)	0.358
ABPR	6 (1.5)	13 (25)	55 (15.9)	0.102
LAVI, ml/m^2^	25 (1.5)	48 (39 ~ 59)	43 (36 ~ 54)	0.051
EDVI, ml/m^2^	0	42 (37 ~ 49)	45 (40 ~ 52)	0.017
ESVI, ml/m^2^	0	16 (14 ~ 19)	18 (15 ~ 21)	0.010
SVI, ml/m^2^	0	25 (22 ~ 30)	27 (24 ~ 31)	0.070
EF, %	0	59.7 (57.9 ~ 63.8)	59.2 (57.0 ~ 62.5)	0.040
IVSTmax, mm	0	23 (21 ~ 29)	24 (20 ~ 27)	0.493
E/e’	10 (1.5)	16.2 (12.9 ~ 21.5)	16.0 (12.9 ~ 20.0)	0.529
Moderate to Severe MR	5 (6.2)	31 (59.6)	143 (41.1)	0.012
Pericardial Effusion	0	9 (17.0)	37 (10.5)	0.166
Pulmonary Hypertension	0	8 (15.1)	18 (5.1)	0.006
Ventricular Aneurysm	0	6 (11.3)	11 (3.1)	0.006
Sarcomere Mutations	0	27 (50.9)	129 (36.6)	0.046
NT-proBNP, pg/ml	0	1934 (905 ~ 2801)	1125 (562.5 ~ 1917.5)	<0.001
RBC	0	4.51 (4.23 ~ 4.79)	4.88 (4.53 ~ 5.26)	<0.001
Hb, g/L	0	134 (124 ~ 148)	150 (138 ~ 161)	<0.001
HCT	0	0.413 (0.382 ~ 0.434)	0.448 (0.415 ~ 0.476)	<0.001
PLT	0	187 (157 ~ 223)	188 (155 ~ 225)	0.735
Creatinine, per umol/L	0	69 (59 ~84)	71 (60 ~84)	0.811
ePVS, ml/g	0	4.40 (3.85 ~ 5.01)	3.70 (3.23 ~ 4.26)	<0.001
Procedure Duration, min	15 (3.7)	72 (58 ~ 89)	80 (60 ~ 104)	0.013

*SBP* Systolic blood pressure, *DBP* Diastolic blood pressure, *HR* Heart rate, *NSVT* Non-sustained ventricular tachycardia, *LVOT-PG* Left ventricular outflow tract pressure gradient, *ABPR* Abnormal blood pressure response which is defined as failure to increase systolic blood pressure by at least 20 mm Hg, *LAVI* Left atrial volume index, *EDVI* Left ventricle end-diastolic volume index, *ESVI* Left ventricle end-systolic volume index, *SVI* Stroke volume index, *EF* Ejection fraction, *IVST* Interventricular septum thickness, *MR* Mitral regurgitation, *NT-proBNP* N-terminal brain natriuretic peptide, *RBC* Red blood cell, *Hb* Hemoglobin, *HCT* Hematocrit, *ePVS* Estimated plasma volume status

**Fig. 1:**
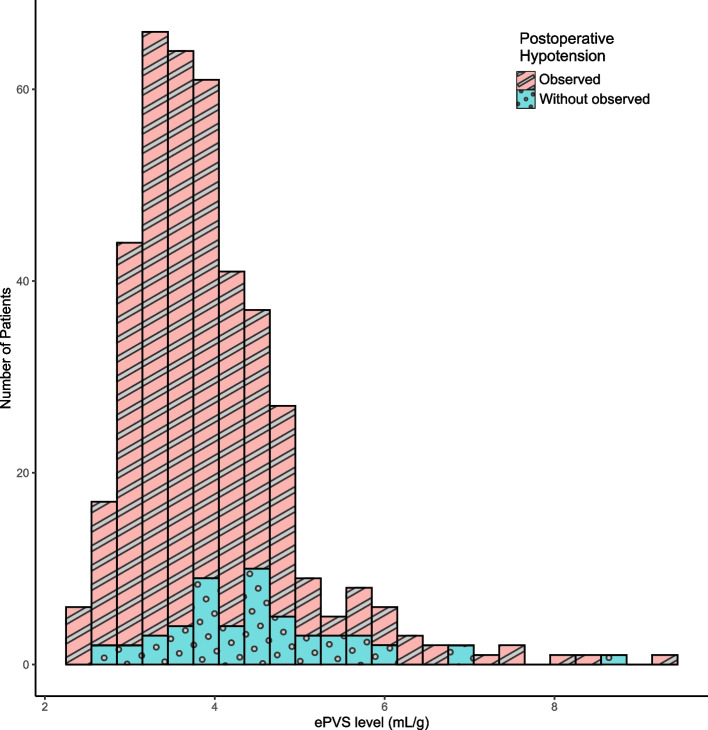
The distribution of ePVS

The distribution of ePVS levels in the 405 patients who underwent PIMSRA is shown in Fig. 1. The median (IQR) of ePVS in overall patients was 3.77 (3.27 ~ 4.40) mL/g. The median (IQR) of ePVS in patients with POH was 4.40 (3.85 ~ 5.01) mL/g, which was significantly higher than those without POH of 3.70 (3.23 ~ 4.26) mL/g (Table 2).

### Association between ePVS and postoperative hypotension

**Fig. 2:**
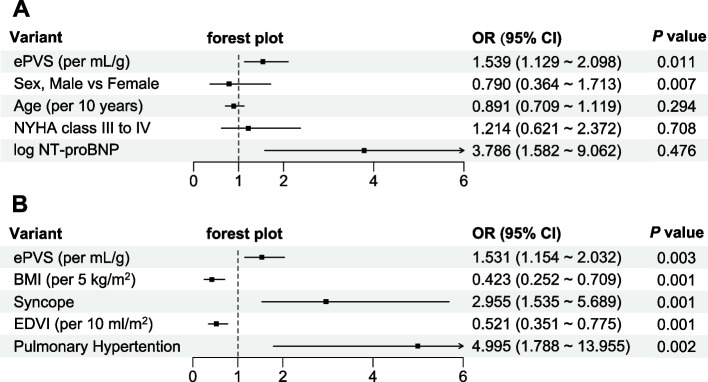
Forest plot for multivariable Logistic regression result. **A** the forest plot of the model derived from ePVS and classic confounding factors; **B** the forest plot of the model derived from ePVS and confounding factors from global optimization search

Among the 405 patients included in this study, 53 (13.1%) patients were observed with symptomatic postoperative hypotension. By univariable Logistic regression analysis, higher ePVS was found to have a significant association with the risk of POH, with the odds ratio (OR) of 1.669 (95% confidence interval [CI] 1.299 ~ 2.144) per mL/g (Supplement Table). After being adjusted by potential confounders, ePVS remained independently associated with POH. In other words, patients with high ePVS exhibited an increased risk for POH (Fig. 2).

### The predictive capacity of ePVS to the postoperative hypotension

**Fig. 3:**
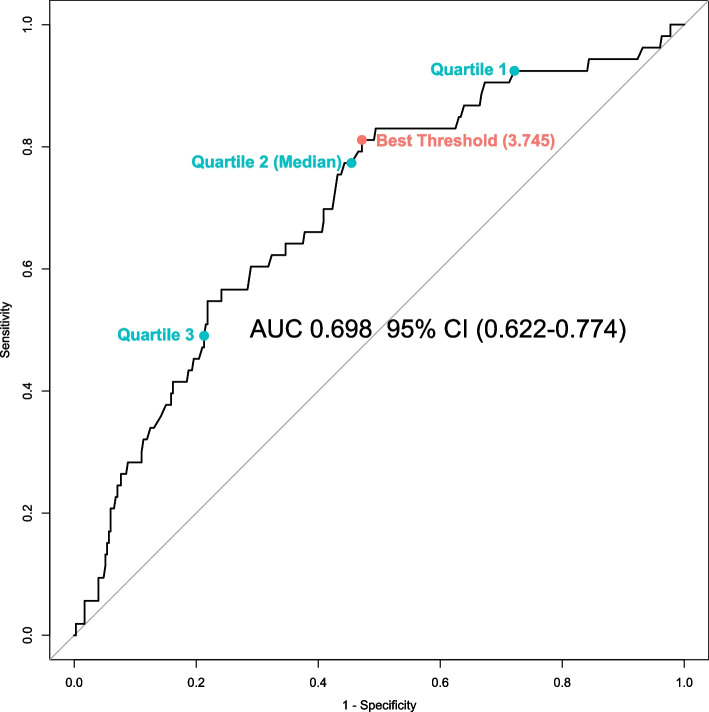
ROC curve for ePVS with postoperative hypotension

Using ePVS to predict POH, the area under the ROC curve (Fig. 3) achieved 0.698 (95% CI, 0.622 to 0.774). It was worth emphasizing that using the best cutoff value, the ePVS achieved an appreciable predictive performance with a sensitivity of 0.811 and a specificity of 0.528.

Using several thresholds including the best cut-off value determined by ROC curve analysis and quartiles, ePVS was transformed into categorical variables which showed different classification performances as shown in Table 3. Using the lower quartile, the sensitivity achieved 92.5%, and instead, using the upper quartile, the specialty achieved 78.7%.

**Table 3:** Association between ePVS and postoperative hypotension

	Value (95% CI)				
	Continuous ePVS	Categorical ePVS 1	Categorical ePVS 2	Categorical ePVS 3	Categorical ePVS 4
Unadjusted OR	1.669 (1.299 ~ 2.144)	4.818 (2.347 ~ 9.890)	4.726 (1.662 ~ 13.442)	4.100 (2.084 ~ 8.066)	3.557 (1.96 ~ 6.454)
Adjusted OR in Model1	1.539 (1.129 ~ 2.098)	4.691 (1.993 ~ 11.042)	4.732 (1.519 ~ 14.738)	3.593 (1.585 ~ 8.142)	2.649 (1.28 ~ 5.479)
Adjusted OR in Model2	1.782 (1.368 ~ 2.323)	5.015 (2.410 ~ 10.438)	4.654 (1.62 ~ 13.373)	4.184 (2.098 ~ 8.347)	3.692 (1.997 ~ 6.826)
Adjusted OR in Model3	1.531 (1.154 ~ 2.032)	3.366 (1.559 ~ 7.265)	3.195 (1.078 ~ 9.472)	2.880 (1.382 ~ 6.001)	2.664 (1.353 ~ 5.247)
Sensitivity	-	0.811 (0.680 ~ 0.906)	0.925 (0.818 ~ 0.979)	0.774 (0.638 ~ 0.877)	0.491 (0.351 ~ 0.632)
Specialty	-	0.528 (0.475 ~ 0.582)	0.278 (0.232 ~ 0.328)	0.545 (0.492 ~ 0.598)	0.787 (0.740 ~ 0.829)
PPV	-	0.206 (0.153 ~ 0.267)	0.162 (0.122 ~ 0.208)	0.204 (0.151 ~ 0.266)	0.257 (0.176 ~ 0.354)
NPV	-	0.949 (0.908 ~ 0.975)	0.961 (0.903 ~ 0.989)	0.941 (0.900 ~ 0.969)	0.911 (0.873 ~ 0.941)

Categorical ePVS 1 was converted by the best threshold in ROC curve analysis. Categorical ePVS 2~4 were converted by the first, second, and third quartile, respectively

Model 1 was derived from ePVS and preassigned confounding factors including sex, age, NYHA III/IV and log-BNP; Model 2 was derived from ePVS and preassigned confounding factors including baseline diuretics usage, procedure duration time and creatinine; Model 3 was derived from ePVS and other confounding factors filtered from a global optimization search

*ePVS* Estimated plasma volume status, *PPV* Positive predictive value, *NPV* Negative predictive value

### Sensitive analysis using the ePVS derived from the Hakim-Kaplan formula

**Fig. 4:**
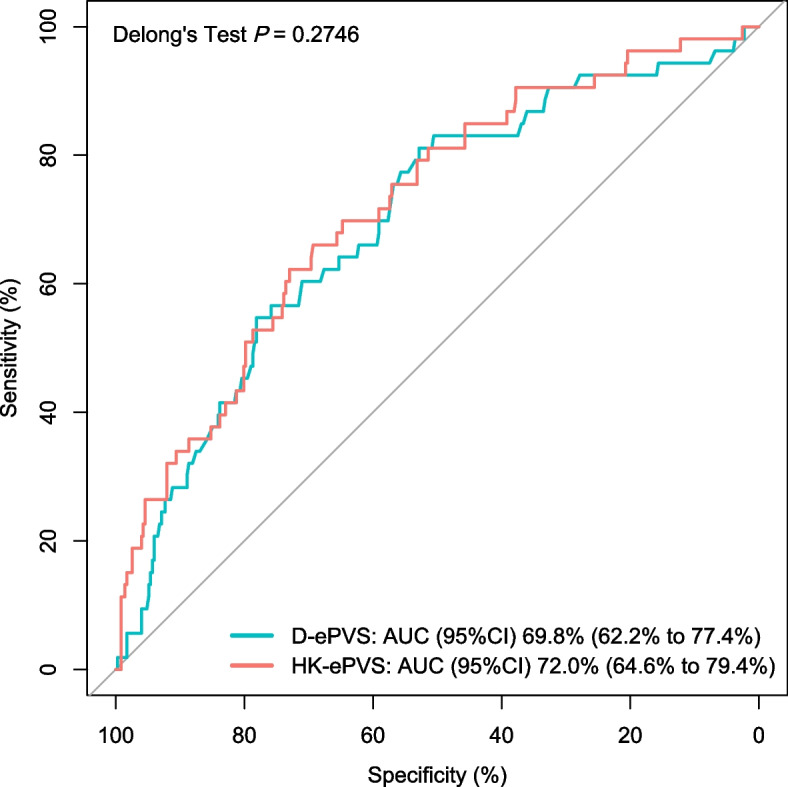
ROC curve for postoperative hypotension with ePVS derived from two formulas. D-ePVS: estimated plasma volume status derived from Duarte formula; HK-ePVS: estimated plasma volume status derived from Hakim-Kaplan formula

Using the ePVS derived from the Hakim-Kaplan formula instead of the Duarte formula, the ePVS showed concordant estimations in univariable and multivariable logistic regression analysis (Table 4). The ePVS using two formulas demonstrated an approximate ROC curve and the two AUROCs were not statistically significant (Fig. 4).

**Table 4:** Association between POH and ePVS derived from two formulas

	Value (95% CI)	
	Duarte ePVS, per mL/g	Hakim-Kaplan ePVS, per 5%
Unadjusted OR	1.669 (1.299 ~ 2.144)	1.618 (1.345 ~ 1.945)
Adjusted OR in Model1	1.539 (1.129 ~ 2.098)	1.528 (1.240 ~ 1.883)
Adjusted OR in Model2	1.782 (1.368 ~ 2.323)	1.673 (1.378 ~ 2.030)
Adjusted OR in Model3	1.531 (1.154 ~ 2.032)	1.503 (1.175 ~ 1.921)

*POH* postoperative hypotension, Duarte ePVS estimated plasma volume status derived from Duarte formula, Hakim-Kaplan ePVS estimated plasma volume status derived from Hakim-Kaplan formula

Model 1 was derived from ePVS and preassigned confounding factors including sex, age, NYHA III/IV, and log-BNP Model 2 was derived from ePVS and preassigned confounding factors including procedure duration time, creatinine, and baseline medication Model 3 was derived from ePVS and other confounding factors filtered from a global optimization search

## Discussion

In this study, we investigated the prevalence of POH and the ePVS in 405 patients with severely symptomatic HOCM who underwent PIMSRA. In this study, results showed that there were 53 (13.1%) patients suffering from POH. The ePVS, which was higher in patients with POH, demonstrated association with the POH. For every 1-unit increase in ePVS, the patient's risk of POH increased by more than 50%, after adjusting for the effects of other confounders. To best of our knowledge, this was the first study that evaluate the ePVS in HOCM patients and investigated its association with POH.

Hypotension and related complications after cardiovascular intervention were common and deleterious, which can cause syncope or shock, leading to irreversible organ damage and even threaten the lives of patients in severe cases [16]. In patients with HOCM, hypotension can lead to exacerbation of LVOT and SAM, which is a key pathophysiological mechanism leading to hemodynamic instability [19, 20]. The timely detection and treatment of POH after PIMSRA is crucial for patients to stably pass through the perioperative period.

The Duarte ePVS provides a method that instantaneously estimate the plasma status [9]. In the largest study to date (data from the NHANS database, involving 42705 participants from 1999-2014), the mean ePVS in the general population was 4.2 ± 0.84 mL/g [10]. Another study recruited 1747 patients diagnosed with HF with preserved ejection fraction and found that the mean ePVS was 4.9 ± 1.0 mL/g [21]. Our research found that ePVS in all HOCM patients was lower compared to that in the general population or patients with heart failure, consistent with the view that HOCM is characterized by reduced intravascular volume and elevated BNP induced by high intracavity pressure in HOCM.

The ePVS was found lower in HOCM patients, but in this cohort the patients observed with POH demonstrated higher ePVS compared with patients without POH. This association may be explained by the relationship between ePVS and cardiovascular function. HOCM patients may develop HF symptoms with progression of disease. The ePVS, an indicator originally designed to quantitatively evaluate congestion in patients with heart failure, is elevated when the HF and fluid retention had developed [22]. Higher ePVS in patients with POH suggested that these patients may have preoperatively suffered from more severe HF and intravascular congestion, leading to lower cardiac reserve and susceptibility to POH, although higher ePVS suggested more sufficient intravascular volume.

Up to now, the ePVS has not been established as a recognized normal range or diagnostic criteria due to the differences of the research population. In particular, there is no large sample study evaluating ePVS in the HCM population. In this study, we found that the median of ePVS in HOCM patients with POH was much higher than that in HOCM patients without POH. The significant difference of ePVS suggested that this index may have some potential and reasonable sensitivity to predict at risk patients for POH.

We performed sensitivity analysis using the Hakim-Kaplan ePVS instead of the Duarte ePVS and found that the two clusters of ePVS were robustly associated with the risk of POH and that both methods might be worthwhile to evaluate the risk of POH in HOCM patients. It's worth noting that the Hakim-Kaplan formula to obtain ePVS requires the dry weight which is often not assessed in patients with cardiovascular diseases. There were some researches suggesting Hakim-Kaplan ePVS calculated from general body weight may also have an association with in-hospital and post-discharge outcomes in decompensated heart failure [4].

This study didn’t exclude the patients who postoperatively received targeted blood pressure management. The ePVS was susceptible to fluid infusion and the postoperative application of fluid infusion and vasopressors may account for the limited predictive performance of ePVS. We hypothesized that changes in ePVS during postoperative management could further add to the prognostic value of ePVS. The perioperative management experience of surgeries including septal reduction therapies suggested that HCM patients may have insufficient cardiac output and need to be monitored during the fluid infusion process to prevent pulmonary edema [23, 24]. Therefore, it is imperative to evaluate the volume status of patients by the ePVS for the perioperative management of patients with hypertrophic cardiomyopathy. Prospective research following a standardized blood sample-collecting method is needed to confirm the application value of ePVS.

This study has several limitations. Firstly, it is based on the retrospective analysis of registry trials and has the inherent limits of a retrospective study, that clinicians or investigators may not capture absolutely accurate information. Second, the sample of blood cell examination is not collected with strict rules, which may bring potential risks of bias. Thirdly, as mentioned before, the postoperative management within all HOCM patients who underwent PIMSRA have individual differences and this may cover the relationship between the ePVS and postoperative hypotension. Fourthly, the lack of routine blood tests during postoperative management precluded the comparison between postoperative ePVS and baseline and this may be an important issue for future research.

## Conclusion

ePVS from Duarte’s formula was associated with increased risk of postoperative hypotension in HOCM patients who underwent PIMSRA. Our findings suggest that ePVS may be a useful variable to assess susceptibility of postoperative hypotension in HOCM patients before PIMSRA and guide postoperative management.

### Supplementary Information

**Supplementary Material 1.**

## Data Availability

The data used during the current study are available on reasonable request from the corresponding author after obtaining the agreement of Institutional Ethics Committee of the Xijing Hospital. All codes and processes used in the current study are available from the corresponding author on reasonable request.

## Abbreviations

HCM: hypertrophic cardiomyopathy
HOCM: hypertrophic obstructive cardiomyopathy
PIMSRA: percutaneous intramyocardial septal radiofrequency ablation
POH: postoperative hypotension
ePVS: estimated plasma volume status
SRT: septal reduction therapy
NYHA: New York Heart Association
HF: heart failure
SBP: systolic blood pressure
DBP: diastolic blood pressure
