# Supplementary material for: Predictive value of estimated plasma volume for postoperative hypotension in percutaneous intramyocardial septal radiofrequency ablation treating for hypertrophic obstructive cardiomyopathy
Source: BMC Cardiovasc Disord. 2024 Mar 22;24:177. doi: 10.1186/s12872-024-03844-9 (PMC10958927; doi:10.1186/s12872-024-03844-9)
Supplement: Supplementary file 1 — Supplementary Material 1. [file 12872_2024_3844_MOESM1_ESM.docx]

**Supplement Table** Results of Univariable Logistic Regression to Postoperative Hypotension

| **Variant** | **OR (95% *CI*)** | ***P* value** |
| --- | --- | --- |
| Sex, Male to Female | 0.36 (0.20 ~ 0.65) | 0.001 |
| Age, year | 1.00 (0.98 ~ 1.02) | 0.943 |
| BMI, kg/m2 | 0.80 (0.73 ~ 0.88) | <0.001 |
| Diabetes Mellitus | 0.86 (0.25 ~ 2.96) | 0.809 |
| Hypertension | 0.39 (0.18 ~ 0.82) | 0.013 |
| Coronary Heart Disease | 0.60 (0.21 ~ 1.76) | 0.354 |
| Family History of HCM | 1.17 (0.61 ~ 2.22) | 0.639 |
| Family History of SCD | 1.92 (0.89 ~ 4.13) | 0.095 |
| ACEI or ARB | 0.46 (0.19 ~ 1.13) | 0.089 |
| *β*-blocker | 0.79 (0.22 ~ 2.82) | 0.721 |
| CCB | 0.58 (0.25 ~ 1.34) | 0.204 |
| Diuretic | 1.01 (0.34 ~ 3.01) | 0.990 |
| Prior SRT | 2.13 (0.67 ~ 6.79) | 0.202 |
| Syncope or presyncope | 3.09 (1.69 ~ 5.65) | <0.001 |
| NYHA III to IV | 1.48 (0.80 ~ 2.74) | 0.209 |
| SBP, mmHg | 0.96 (0.94 ~ 0.98) | 0.001 |
| DBP, mmHg | 0.95 (0.92 ~ 0.99) | 0.008 |
| HR, bpm | 1.00 (0.97 ~ 1.03) | 0.899 |
| NSVT | 1.13 (0.50 ~ 2.53) | 0.774 |
| Atrial Fibrillation | 1.17 (0.39 ~ 3.52) | 0.783 |
| LVOTPG at rest, mmHg | 1.00 (1.00 ~ 1.01) | 0.280 |
| LVOTPG provoking, mmHg | 1.00 (0.99 ~ 1.00) | 0.539 |
| ABPR | 1.75 (0.87 ~ 3.49) | 0.116 |
| LAVI, ml/m^2^ | 1.02 (1.00 ~ 1.03) | 0.044 |
| EDVI, ml/m^2^ | 0.96 (0.92 ~ 0.99) | 0.009 |
| ESVI, ml/m^2^ | 0.90 (0.84 ~ 0.97) | 0.004 |
| SVI, ml/m^2^ | 0.94 (0.89 ~ 1.00) | 0.048 |
| EF, % | 1.08 (1.01 ~ 1.15) | 0.034 |
| IVSTmax, mm | 1.01 (0.96 ~ 1.07) | 0.666 |
| E/e’ | 1.02 (0.98 ~ 1.07) | 0.301 |
| Moderate to Severe MR | 1.74 (0.79 ~ 3.85) | 0.171 |
| Pericardial Effusion | 3.30 (1.36 ~ 8.03) | 0.009 |
| Pulmonary Hypertension | 3.96 (1.40 ~ 11.20) | 0.010 |
| Mutations in Sarcomere Genes | 1.80 (1.00 ~ 3.21) | 0.048 |
| log BNP | 4.33 (1.90 ~ 9.89) | <0.001 |
| RBC | 0.24 (0.13 ~ 0.44) | <0.001 |
| Hb, g/L | 0.97 (0.95 ~ 0.98) | <0.001 |
| HCT | 0.00 (0.00 ~ 0.00) | <0.001 |
| PLT | 1.00 (0.99 ~ 1.00) | 0.670 |
| ePVS, ml/g | 1.67 (1.30 ~ 2.14) | <0.001 |
